# Urethral Calculus1Presented at the Fourteenth Annual Meeting of the Iowa State Veterinary Medical Association, Des Moines, Iowa, February 11 and 12, 1902.

**Published:** 1902-04

**Authors:** E. G. Marten

**Affiliations:** Schaller, Iowa


### URETHRAL CALCULUS.[1]

By E. G. Marten, M.D.C.,
SCHALLER, IOWA.

On an October morning Mr. J. B. Harris brought to me a horse that he said could not urinate. On examination I found the bladder distended with urine and the urine dripping from the penis. On trying to pass the catheter I found it difficult, but finally succeeded. On taking out the catheter some small stones followed, but the big ones remained, and as I could not reach them with my forceps I decided to operate. It being Sunday, Mr. Harris wished me to wait until the next day. The following day I passed the catheter and withdrew the urine, and then prepared for the operation. The horse was cast as for castration. The part was washed with castile soap and water and with a 1 to 20 solution of Pearson's creolin. The skin was then rendered tense by the thumb and finger of the left hand, and an incision was made with the scalpel in the median line in the perineal region; then with a large splinter forceps I extracted twenty-five calculi, from the size of a large pea to the size of a large walnut. After washing thoroughly and putting three sutures into the incision in the urethra and one in the skin the animal was allowed to rise. The wound healed nicely, and the animal made a complete recovery.

[1] Presented at the Fourteenth Annual Meeting of the Iowa State Veterinary Medical Association, Des Moines, Iowa, February 11 and 12, 1902.

### Discussion.

Dr. S. H. Bauman said that on May 28, 1901, a roan mare, about eight years old, was brought to his barn. The history as given by the owner was that she was unable to hold her urine, and that he had owned her for a year or more, during which she worked well every day, and was always in good flesh and easily kept. Her coat was always glossy, she had good life and ambition, and looked to be in the best of health. The owner told me he had to wash her tail every day or so, on account of the disagreeable odor of urine. I proceeded to make an examination. I inserted my hand into the vagina, and felt a tumor in the bladder as large as my two fists. I inserted a probe, but could feel no calculus. I also inserted a pair of lithotomy forceps and probed with them. I then told the owner that all I could do was to cut on the tumor and find what was encysted, and that probably we could effect a cure or better the condition of the mare. With his consent I proceeded to do so. I used creolin solution and thoroughly cleansed the parts. I then took a short castrating knife with a hooked blade, and made an incision about four inches long, starting on a line just anterior to the meatus urinarius, and inserted my hand through the opening. I cut down on the tumor and found the calculus encysted. I carefully dissected around the calculus and removed it as well as another smaller one which lay in the same sac. The growth around the calculus was about an inch to an inch and a half thick, and resembled superfluous granulations as seen in an open wound. It was very vascular, and the hemorrhage was diffuse. I had a loss of perhaps two quarts of blood during the removal. The calculus is almost eleven inches at its greatest circumference, four inches at its greatest diameter, and weighs almost thirteen ounces. I now exhibit it to you. There are four or five more of the same nature in the bladder of this same mare, but I wanted the mare to live, so stopped with this one. In conclusion, will say that I dressed the wound and let the mare stand for an hour. Her owner drove her home the same day, a distance of ten miles, where she has worked right along ever since. The only difference seen or benefit derived from the operation is that she is able to retain her urine a little longer than before. Probably by the time of our next meeting I will be able to give you more on this same subject, as I expect to operate again this spring.

